# Prognostic prediction by hypermetabolism varies depending on the nutritional status in early amyotrophic lateral sclerosis

**DOI:** 10.1038/s41598-021-97196-5

**Published:** 2021-09-09

**Authors:** Ryutaro Nakamura, Mika Kurihara, Nobuhiro Ogawa, Akihiro Kitamura, Isamu Yamakawa, Shigeki Bamba, Mitsuru Sanada, Masaya Sasaki, Makoto Urushitani

**Affiliations:** 1 Department of Neurology, Shiga University of Medical Science, Seta-Tsukinowa-cho, Otsu, Shiga 520-2192 Japan; 2 Division of Clinical Nutrition, Shiga University of Medical Science, Seta-Tsukinowa-cho, Otsu, Shiga 520-2192 Japan

**Keywords:** Diseases, Health care, Medical research, Predictive markers, Prognostic markers, Neurology, Amyotrophic lateral sclerosis

## Abstract

To examine whether hypermetabolism could predict the prognosis of early amyotrophic lateral sclerosis (ALS) patients with differing nutritional profiles. This single-center, retrospective study examined the prognosis of ALS patients with hypermetabolism in relation to their nutritional status at hospitalization. The metabolic state was estimated by the ratio of measured resting energy expenditure (mREE) to lean soft tissue mass (LSTM) (mREE/LSTM), wherein patients with ratios ≥ 38 were defined as hypermetabolic. Malnutrition was defined as %ideal body weight < 0.9. Forty-eight patients were enrolled in this study. The hypermetabolic group had shorter survival in the normal-weight group but more prolonged survival in the malnutrition group. Multiplication of nutritional and metabolic factors, such as [(body mass index (BMI) − 19.8) × (mREE/LSTM − 38)], designated as BMI-muscle metabolism index (BMM index), successfully predicted the prognosis in the group with a high BMM index (≥ 1), which showed shorter survival and a faster rate of weight loss and functional decline. Multivariate analysis using the Cox model showed high BMM index was an independent poor prognostic factor (hazard ratio: 4.05; *p* = 0.025). Prognostic prediction by hypermetabolism varies depending on the nutritional status in ALS, and the BMM index is a consistent prognostic factor.

## Introduction

Amyotrophic lateral sclerosis (ALS) is a fatal neurodegenerative disorder characterized by progressive degeneration of the upper and lower motor neurons, with a median survival time of two to four years^1^. ALS is caused by diverse pathomechanisms, including genetic defects and dysregulated cellular machinery, such as proteostasis, RNA metabolism, mitochondria, excitotoxicity, and neuroinflammation^2–5^. Although disease-modifying drugs are underway, the therapeutic efficacy of currently available drugs––riluzole and edaravone––is unfortunately limited.

Evidence regarding the significance of non-pharmacological treatment is accumulating, indicating that it can improve the quality of life and the progression or survival of ALS. Early introduction of non-invasive positive pressure ventilation (NPPV), especially with cough assist devices, is reported to prolong the lifespan of ALS^6^. In addition, a lower body mass index (BMI) less than 18.5 at the first visit to the neurologists has been well-recognized as a poor prognostic factor, and a high-calorie diet effectively slows ALS progression, especially in rapid progressors^7,8^. Surprisingly, in rapidly progressing ALS, a fat-dominant high-calorie diet inhibits the elevation of serum neurofilament-L (NFL), a compelling biomarker of neuronal damage^7^. Despite these lines of clinical evidence, the mechanism by which the reduction of body weight or the intake of a high-calorie diet confers protection in ALS is not well known.

Previous studies have shown that metabolism and nutritional status play an essential role in the progression of ALS. Hypermetabolism^9–11^, low body fat percentage^12^, and low fat-free mass^13^ were poor prognostic factors, as confident as serum creatine phosphokinase (CPK), creatinine, albumin, and ferritin, as previously reported^1,3,14,15^. Patients with weight loss had shorter survival time, and weight loss also affected functional outcomes after tracheostomy^16,17^. Although not evident, subthalamic dysfunction, dysregulated autophagy, impaired mitochondrial function, and excitotoxicity^4,18^ are implicated in the hypermetabolism of ALS patients^9^, which leads to weight loss^19^.

Identifying the energy source is essential when analyzing metabolism. ALS patients have impaired glucose tolerance at the early stage, and free fatty acids are higher in such patients than in those with normal glucose tolerance^20^. It was reported that the energy production pathway changed from glycolytic to lipid metabolism during starvation in ALS mice, in a process called “fuel switch”^4^. This indicates that lipids may be the primary fuel in patients with malnutrition as well as ALS mice, although no studies examined changes in metabolism in response to the nutritional status of humans. Elevated levels of total cholesterol and low-density lipoprotein cholesterol (LDL), and a higher LDL/ high-density lipoprotein cholesterol (HDL) ratio has been associated with longer survival^21,22^, which might indicate the benefit of lipid metabolism. Therefore, we retrospectively examined our institutional cohort to test the hypothesis that the impact of hypermetabolism on survival might differ depending on the nutritional status.

## Materials and methods

### Patients and ethical considerations

This is a single-center retrospective study of ALS patients admitted to Shiga University of Medical Science Hospital from March 2018 to February 2021 for close investigation, the introduction of therapeutic drugs and NPPV, or percutaneous endoscopic gastrostomy (PEG). We collected the data from the first admission in this period. All patients fulfilled the revised El Escorial criteria for probable or definite ALS^23^ and underwent indirect calorimetry. We excluded patients who were younger than 49 years old, who had used mechanical ventilation, including NPPV, and who had experienced PEG until this admission to avoid the influence of metabolism analysis. This study was approved by the Ethics Committee of Shiga University of Medical Science Hospital (approval no.: R2020-171). We applied Opt-out method and obtained informed consent on this study, which have been performed in accordance with the ethical standards laid down in the 1964 Declaration of Helsinki and its later amendments. The participants have consented to the submission of this study to the journal.

### Clinical parameters

Nutritional status was estimated by BMI at this admission, and we calculated the percent ideal body weight (%IBW), using the following formula: %IBW = body weight at this admission/22 × ([patient’s height in meters] ^2). We defined malnutrition as %IBW < 0.9, according to a previous report^24^. We evaluated the monthly rate of BMI decline since onset to this admission (ΔBMI), using the following formula: (BMI at this admission − premorbid BMI)/(time since onset in months). To assess the disease progression, we calculated the monthly decline rate of the Revised Amyotrophic Lateral Functional Rating Scale (ALSFRS-R) score from onset to this admission (ΔALSFRS-R), using the following formula: (48 − ALSFRS-R at this admission)/(time from onset to this admission in months)^25^. We also evaluated the following factors: age, sex, body region affected at the onset, excess weight loss (more than 10% compared to the body weight before the illness), and time since the first visit. To assess the influence of treatment on survival time, we collected the data of using NPPV and PEG tube feeding.

### Energy metabolism and body composition

We collected data on the resting energy expenditure (REE) and respiratory quotient (RQ), which were measured by indirect calorimetry (Aeromonitor AE310S, Minato Medical Science Co., Ltd.). Indirect calorimetry was performed for 10 min in the morning after the patient had fasted overnight and rested in the supine position on a bed for 30 minutes^26^. We calculated the RQ as carbon dioxide production divided by oxygen uptake (VCO2/VO2) and calculated the measured REE (mREE; kcal) using the Weir equation without urinary nitrogen^27^. We estimated the predicted REE (pREE; kcal) via the Harris-Benedict Eq. ^28^. To evaluate body composition, we collected the data of lean soft tissue mass (LSTM; kg), body fat percentage (%), and skeletal muscle index (SMI; kg/m^2^), which were measured by bioelectrical impedance analysis with a body composition analyzer (InBody S10; InBody, Tokyo, Japan). The bioelectrical impedance analysis was performed concurrently with the indirect calorimetry.

As for the definition of hypermetabolism, we used mREE/LSTM rather than mREE/pREE^9,10^ because pREE tends to be overestimated in Japanese people^29^. Although previous reports used mREE/fat-free mass as a factor associated with hypermetabolism^9,30,31^, we replaced fat-free mass with LSTM expecting to get a more accurate evaluation of metabolism per lean tissue because LSTM could discriminate lean tissue from bone quantity and be effective for assessing skeletal muscle mass^32,33^.

We defined hypermetabolism as mREE/LSTM ≥ 38 kcal/kg, considering the percentage of patients with hypermetabolism. The number of patients with mREE/LSTM ≥ 38 was 20/46 (43.4%) in our cohort, while the number of patients with hypermetabolism was 24/58 (41.3%) in the study by Steyn^10^, which confirmed the validity of the cut-off value. We defined the high body fat percentage as more than 25% in men and 30% in women. For the RQ, we determined the cut-off value as 0.85, because it is 0.7 for fat and 1.0 for carbohydrates^34^.

### Other biomarkers

We collected the following data of respiratory function tests measured by spirometry: %peak expiratory flow (%PEF; %), and %vital capacity (%VC; %). We extracted the following data of blood biomarkers on the day closest to that in which indirect calorimetry was performed: LDL; mg/dl, and fasting blood sugar (FBS; mg/dl). We defined high LDL as LDL ≥ 100 according to a previous report^22^.

### Endpoints

We defined endpoints as the time of death or tracheotomy and survival time as the duration from onset to endpoints or censoring time. The censoring time for the follow-up was the end of February 2021.

### Statistical analysis

We classified patients by metabolic status dependent on their nutritional status. We used Fisher’s exact test or Mann–Whitney U test for data comparison between the hypermetabolic group and normally metabolic group depending on nutritional status. To estimate the absolute risks of hypermetabolism on survival depending on nutritional status, we used Kaplan–Meier curves and the log-rank test. Similarly, we determined high mREE/pREE as > 1.1^9^ to validate it as a prognostic factor in different nutritional statuses. Furthermore, excess weight loss, body fat percentage, and LDL were assessed for stratification by using the Kaplan–Meier curves and the log-rank test. Kaplan Myer curves start below 100% survival when the endpoint occurs within one month after this admission. To investigate the association between the demographics and body fat percentage, mREE/LSTM, mREE/pREE, or RQ, we used the Spearman rank-correlation method. Variables are summarized as the median and interquartile range for continuous variables or frequency for categorical variables; missing values were excluded. Statistical analyses were two-sided, and *p* < 0.05 was considered significant. All analyses were performed by using EZR version 1.53 (Saitama Medical Center, Jichi Medical University, Japan)^35^. To create figures, we used EZR and Microsoft Excel version 2016.

## Result

### Clinical characteristics and outcomes

Forty-eight patients were included in this study (25 men; 23 women); body composition data were lacking in two male patients. The median mREE/LSTM of the male group was 35.9 kcal/kg (interquartile range 34.4 to 39.6), and that of the female group was 36.9 kcal/kg (interquartile range 34.5 to 42.3), which were not significantly different (*p* = 0.50). We classified patients by the metabolic state depending on nutritional status; the clinical profiles of which were summarized in Table 1. Age and sex were not significantly different between the hypermetabolic and normal metabolic groups in the normal-weight group and the malnutrition group. The hypermetabolic normal-weight group had the following characteristics: higher mREE/pREE (*p* = 0.044), and lower SMI (*p* = 0.028). On the other hand, the hypermetabolic malnutrition group had a longer time since onset (*p* = 0.048), higher FBS (*p* = 0.023), and higher %PEF (*p* = 0.046).

**Table 1:** Clinical characteristics and biomarkers.

Metabolism (n)	All (48)	%IBW ≥ 0.9		p*	%IBW < 0.9		p*
		Hyper (11)	Normal (18)		Hyper (9)	Normal (8)	
Age (years)	71 [65, 75]	73 [67, 75]	69 [61, 75]	0.62	71 [67, 74]	69 [63, 74]	0.81
Sex (female)	23/48 (48%)	5/11	7/18	1	5/9	6/8	0.62
Premorbid BMI	23.2 [20.1, 25.0]	24.5 [22.8, 25.4]	23.9 [23.32 26.5]	0.77	19.0 [18.6, 20.2]	20.0 [19.1, 22.4]	0.114
BMI at this admission	21 [19, 23]	22 [21, 23]	22 [21, 25]	0.64	19 [18, 19]	19 [17, 19]	0.96
ΔBMI (/M)	− 0.13 [− 0.25, 0.00]	− 0.20 [− 0.26, − 0.06]	− 0.03 [− 0.23, 0.00]	0.26	− 0.01 [− 0.19, 0.00]	− 0.04 [− 0.55, − 0.08]	0.114
Excess weight loss (yes)†	12/48 (25%)	3/11	5/18	1	1/9	3/8	0.29
Time since onset (M)	15 [8, 30]	10 [8, 15]	17 [11, 34]	0.065	24 [12, 31]	9 [8, 13]	**0.048**
Time since the first visit	2 [1, 5]	1 [1, 3]	1 [1, 6]	0.47	1 [0, 18]	2 [1, 4]	0.92
ALSFRS-R	39 [35, 43]	36 [32, 44]	40 [34, 45]	0.70	38 [36, 43]	41 [40, 42]	0.74
ΔALSFRS-R	0.59 [0.27, 0.91]	0.60 [0.34, 1.5]	0.42 [0.18, 0.77]	0.22	0.66 [0.19, 0.73]	0.66 [0.48, 0.91]	0.44
Bulbar type (yes)	12/48 (25%)	2/11	3/18	1	2/9	5/8	0.153
FBS (mg/dl) (n)	98 [86, 107] (40)	97 [91, 106] (10)	98 [89, 110] (16)	0.90	104 [89, 115] (6)	83 [83, 85] (6)	**0.023**
LDL (mg/dl) (n)	109 [96, 132] (40)	100 [85, 104] (10)	108 [92, 127] (15)	0.47	143 [117, 155] (8)	116 [101, 138] (6)	0.30
%PEF (%) (n)	77 [61, 91] (46)	70 [59, 88] (10)	90 [70, 103] (18)	0.175	85 [69, 99] (8)	65 [54, 75] (7)	**0.046**
%VC (%) (n)	87 [78, 94] (46)	85 [67, 95] (10)	91 [84, 100] (18)	0.175	81 [79, 90] (8)	81 [61, 89] (7)	0.72
Body fat (%) (n)	31.7 [26.2, 37.1] (46)	34.9 [30.4, 43.7]	30.3 [27.1, 36.4]	0.20	32.1 [27.7, 37.3]	27.2 [22.4, 31.0]	0.114
LSTM (kg) (n)	34 [29, 40] (46)	33 [28, 40]	38 [33, 44]	0.101	29 [24, 38]	31 [29, 35]	0.61
mREE/LSTM (n)	36.4 [34.4, 40.5] (46)	40.6 [39.4, 42.1] (11)	34.4 [33.5, 34.9] (18)	< 0.001	41.7 [39.5, 43.2] (9)	34.5 [34.1, 35.6] (8)	< 0.001
mREE/pREE	1.07 [1.00, 1.16]	1.12 [1.05, 1.25]	1.07 [0.99, 1.08]	**0.044**	1.08 [1.00, 1.15]	1.02 [0.97, 1.06]	0.28
RQ < 0.85 (yes)	29/48 (60%)	6/11	10/18	1	8/9	4/8	0.131
SMI (kg/m^2^) (n)	5.8 [4.7, 6.4] (46)	5.6 [4.5, 6.2]	6.4 [5.9, 7.1]	**0.028**	4.7 [3.7, 5.8]	5.1 [4.6, 5.8]	0.56
PEG (yes)	25/48 (52%)	8/11	5/18	**0.027**	7/9	4/8	**0.035**
NPPV (yes)	27/48 (57%)	7/11	9/18	0.71	7/9	4/8	0.34

Bold font was used to strengthen the statistical significance.

Data presented as median [Interquartile range]. (n) shows the number of patients in factors that have missing values. Due to missing values, the sum of the numbers in each group does necessarily equal the total number.

*IBW* Ideal body weight, *BMI* Body mass index, *ALSFRS-R* Revised amyotrophic lateral functional rating scale, *FBS* Fasting blood sugar, *LDL* Low-density lipoprotein cholesterol, *PEF* Peak expiratory flow, *VC* Vital capacity, *LSTM* Lean soft tissue mass, *REE* Resting energy expenditure, *mREE* Measured REE, *pREE* Predicted REE, *RQ* Respiratory quotient, *SMI* Skeletal muscle index, *PEG* Percutaneous endoscopic gastrostomy, *NPPV* Non-invasive positive pressure ventilation.

*p value is based on Fisher’s exact test or Mann–Whitney U test. †Excess weight loss means more than 10% compared to the body weight before the illness.

**Figure 1:**
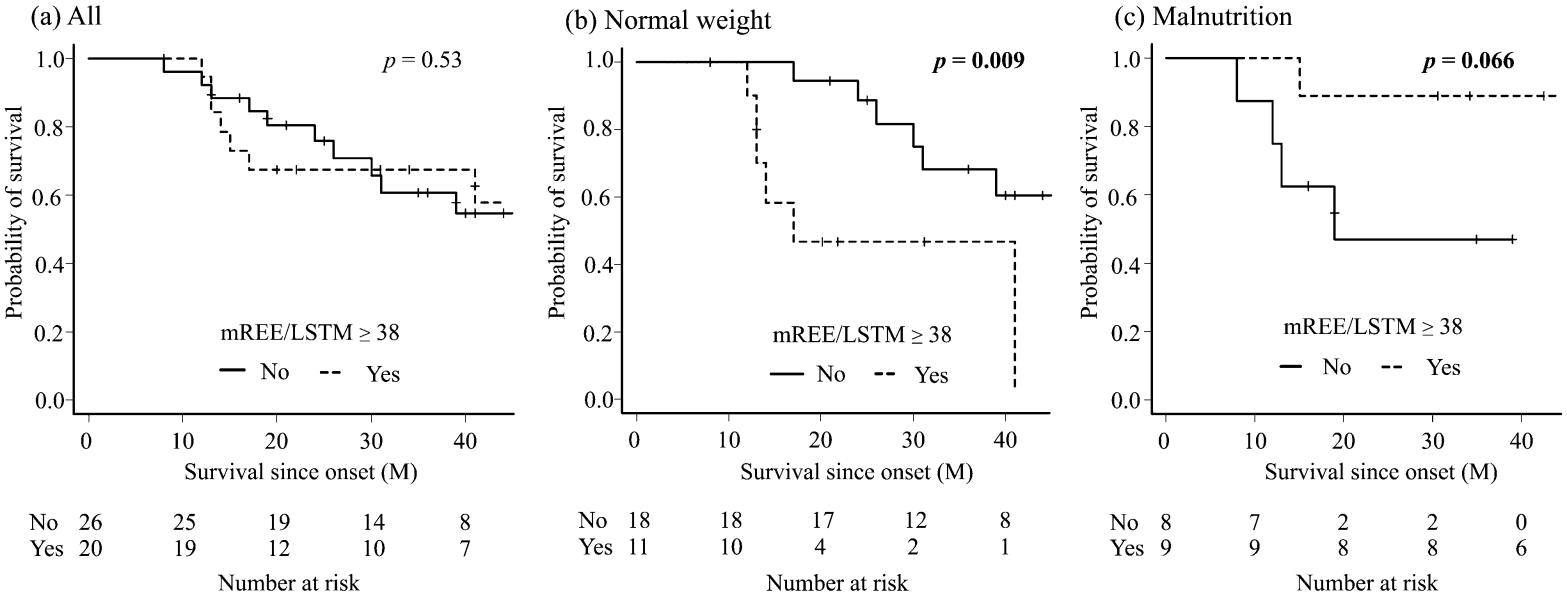
Comparison of survival rate between the hypermetabolic group and the normal metabolic group in all patients (**a**), the normal-weight group (**b**), the malnutrition group (**c**). Kaplan–Meier analyses and the log-rank test showed there was no difference in all patients (*p* = 0.53); however, the hypermetabolic group had a significantly shorter survival time in the normal-weight group (*p* = 0.009) and longer survival time in the malnutrition group (*p* = 0.066), although the difference was not significant.

**Figure 2:**
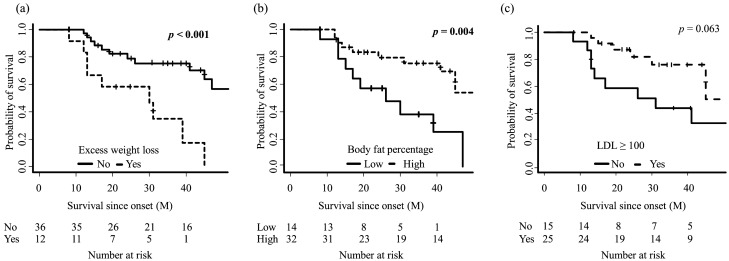
Comparison of survival rate between amyotrophic lateral sclerosis patients stratified by excess weight loss (**a**), body fat percentage (**b**), and low-density lipoprotein cholesterol (LDL) (**c**). Kaplan–Meier analyses and the log-rank test showed patients with excess weight loss (*p* < 0.001) and low body fat percentage (*p* = 0.004) had significantly shorter survival time. Patients with low LDL also had shorter survival time, although the difference was not significant (*p* = 0.063).

The log-rank test showed that neither metabolic state nor nutrition state alone was a statistically independent prognostic factor (*p* = 0.53, *p* = 0.99, respectively). The hypermetabolic group had a shorter survival time in normal-weight patients (*p* = 0.009) but showed a trend of longer survival time in the malnutrition group (*p* = 0.066) (Fig. 1), which was also seen in the log-rank test for time since this admission (Supplemental Fig. 1). Of note, mREE/pREE alone was not a statistically significant prognostic factor among all patients (*p* = 0.43), the normal-weight group (*p* = 0.70), or the malnutrition group (*p* = 0.73) (Supplemental Fig. 2). As for other risk factors, high body fat and excess weight loss were good (*p* = 0.004) and poor (*p* < 0.001) prognostic factors for survival time (Fig. 2). Patients with low LDL had a propensity toward shorter survival, although the difference was not significant (*p* = 0.063). In the log-rank test for the survival time since this admission, patients with excess weight loss (*p* = 0.007) and low LDL (*p* < 0.001) had shorter survival, and patents with low body fat showed similar trend (*p* = 0.106) (Supplemental Fig. 3).

**Figure 3:**
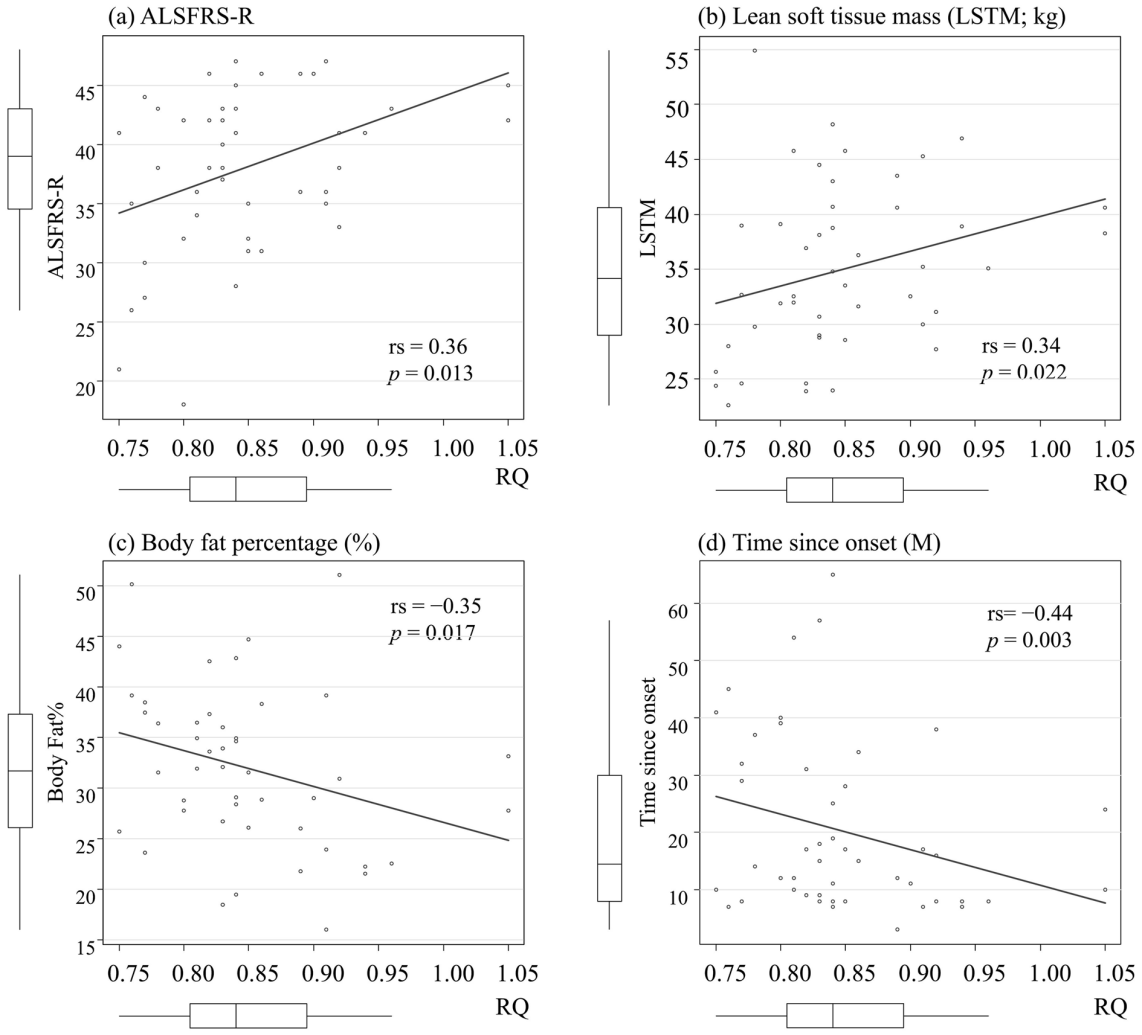
The association between demographics and respiratory quotient (RQ). RQ was significantly correlated with ALSFRS-R (**a**), lean soft tissue mass (**b**), body fat percentage (**c**), and time since onset (**d**). Rs means Spearman’s rank-correlation coefficient. *p* value is based on Spearman’s test.

We examined demographics associated with RQ, mREE/LSTM, mREE/pREE, or body fat percentage. RQ was associated with ALSFRS-R (*p* = 0.013), time since onset (*p* = 0.003), body fat percentage (*p* = 0.017), and LSTM (*p* = 0.022) (Fig. 3). mREE/LSTM was positively associated with body fat percentage (rs = 0.36, *p* = 0.015), and negatively with BMI (rs =  − 0.29, *p* = 0.048), and LSTM (rs =  − 0.46, *p* = 0.002) (Supplemental Fig. 4). mREE/pREE was positively associated with LSTM (rs = 0.44, *p* = 0.002), and negatively with body fat percentage (rs =  − 0.42, *p* = 0.004). mREE/pREE was not associated with BMI (rs = 0.00, *p* = 0.99). Body fat percentage was positively correlated with BMI (rs = 0.38, *p* = 0.009) and negatively with LSTM (rs =  − 0.54, *p* < 0.001).

### BMM index

The discrepant trends in survival times between the hypermetabolic patients in the normal-weight and malnutrition groups led us to estimate the influence of hypermetabolism on survival and nutritional status. We tried to obtain the new evaluation plan named “BMI-muscle metabolism (BMM) index” via the following equation: (BMI − 19.8) × (mREE/LSTM − 38). We subtracted 19.8 from BMI because %IBW < 0.9 means BMI < 19.8. Since a BMI of 19.8 and a mREE/LSTM of 38 are normal values, the BMM index increases when both are more or less than average. We thus tested whether a high BMM index predicted a poor prognosis.

**Figure 4:**
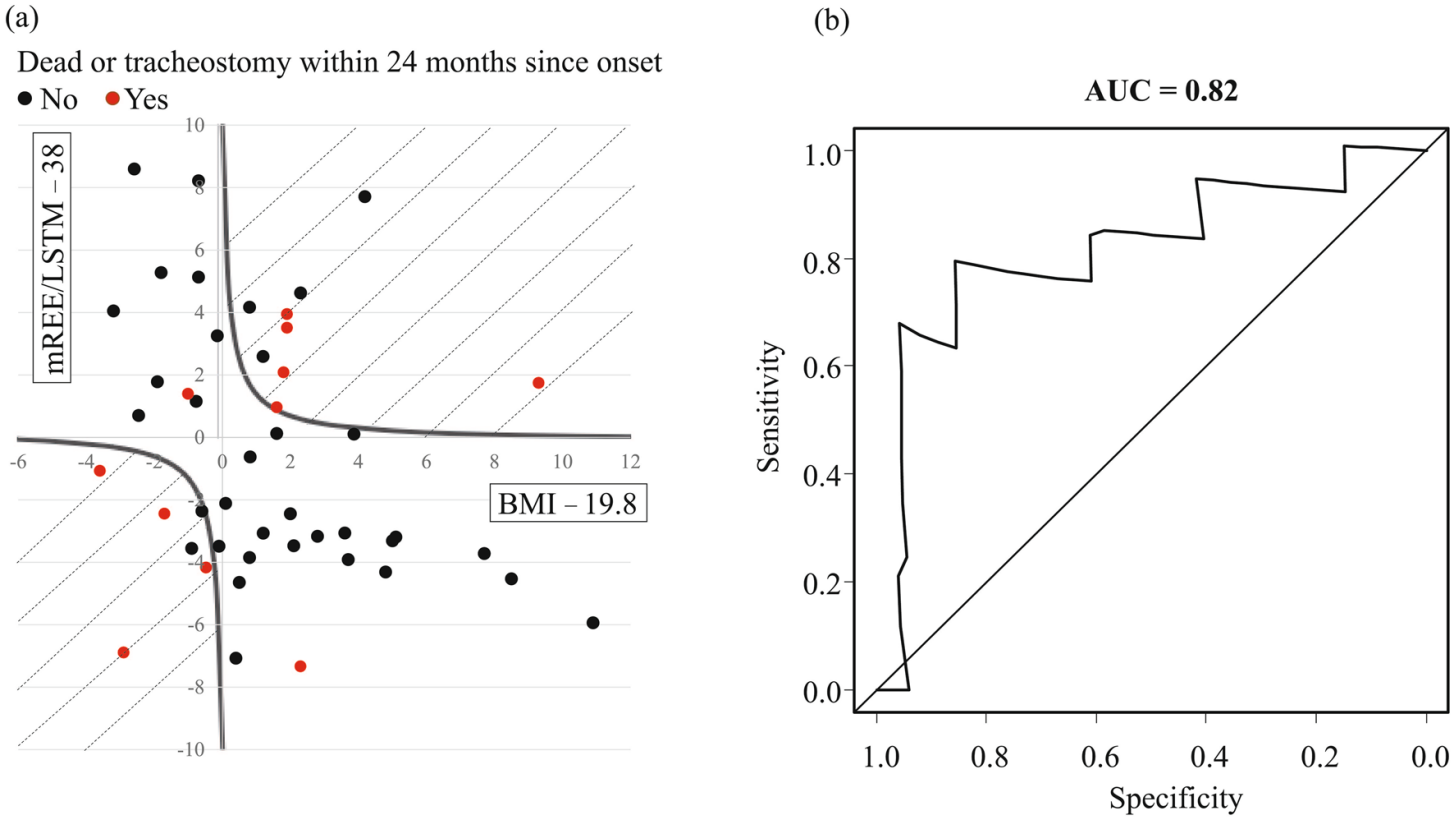
The prognostic value of BMI-muscle metabolism index (BMM Index). Patients who died or underwent tracheostomy within 24 months from onset are presented as red circles, and the remaining patients are presented as black circles (**a**). The shaded area shows the body mass index-muscle metabolism (BMM) index ≥ 1 (**a**). Time-dependent receiver operating characteristic curve analysis shows that the BMM index strongly predicts the incidence of endpoints within two years from the onset (area under the curve = 0.82) (**b**).

We determined the cut-off value of BMM Index with time-dependent receiver operating characteristic (ROC) curves^36^, which confirmed that the BMM index predicted the incidence of endpoints within two years from the onset (AUC = 0.82), with a sensitivity of 0.77, and a specificity of 0.88 when the BMM index was higher than one (Fig. 4). Therefore, we determined the cut-off value of the BMM index as one and classified patients by this cut-off value into two groups. We used Fisher’s exact test and the Mann–Whitney U test to compare the two groups and summarized demographics and biomarkers (Table 2).

**Table 2:** Demographics and biomarkers.

BMM index	< 1	≥ 1	p*
n	31	15	
Age (years)	69 [62, 74]	73 [68, 75]	0.23
Sex (female)	15/31 (48%)	8/15 (53%)	1
BMI	21 [19, 23]	21 [19, 22]	0.57
ΔBMI(/M)	− 0.02 [− 0.19, 0.00]	− 0.25 [− 0.31, − 0.16]	**0.004**
Excess weight loss (yes)^†^	6/31 (19%)	6/15 (40%)	0.165
Time since onset (M)	17 [12, 32]	8 [8, 11]	**0.005**
ALSFRS-R	41 [36, 45]	38 [34, 42]	0.196
ΔALSFRS-R	0.44 [0.16, 0.72]	0.88 [0.59, 1.5]	**0.003**
Bulbar type (yes)	6/31 (19%)	6/15 (40%)	0.165
LDL (mg/dl) (n)	122 [102, 143] (27)	98 [91. 103] (12)	**0.011**
%VC (%) (n)	90 [82, 98] (30)	78 [55, 86] (14)	**0.002**
mREE/pREE	1.07 [1.00, 1.11]	1.06 [1.00, 1.21]	0.66
RQ < 0.85 (yes)	20/31 (65%)	8/15 (53%)	0.53
High-fat (yes)	22/31 (71%)	10/15 (67%)	1
Lean soft tissue mass (kg)	36 [31, 42]	31 [28, 37]	0.143
SMI (kg/m^2^)	6.0 [5.2, 6.8]	5.5 [4.5, 6.1]	0.073
PEG (yes)	14/31 (45%)	10/15 (67%)	0.22
NPPV (yes)	20/31 (65%)	7/15 (47%)	0.22
Endpoint (yes)	11/31 (35%)	10/15 (67%)	0.063

Bold font was used to strengthen the statistical significance.

Data presented as median [Interquartile range]. (n) shows the number of patients in factors that have missing values.

*BMM* BMI-muscle metabolism, *BMI* Body mass index, *ALSFRS-R* Revised amyotrophic lateral functional rating scale, *LDL* Low-density lipoprotein cholesterol, *VC* Vital capacity, *REE* Resting energy expenditure, *mREE* Measured REE, *pREE* Predicted REE, *RQ* Respiratory quotient, *SMI* Skeletal muscle index, *PEG* Percutaneous endoscopic gastrostomy, *NPPV* Non-invasive positive pressure ventilation.

*p value is based on Fisher's exact test or Mann–Whitney U test.

^†^Excess weight loss means more than 10% compared to the body weight before the illness.

**Figure 5:**
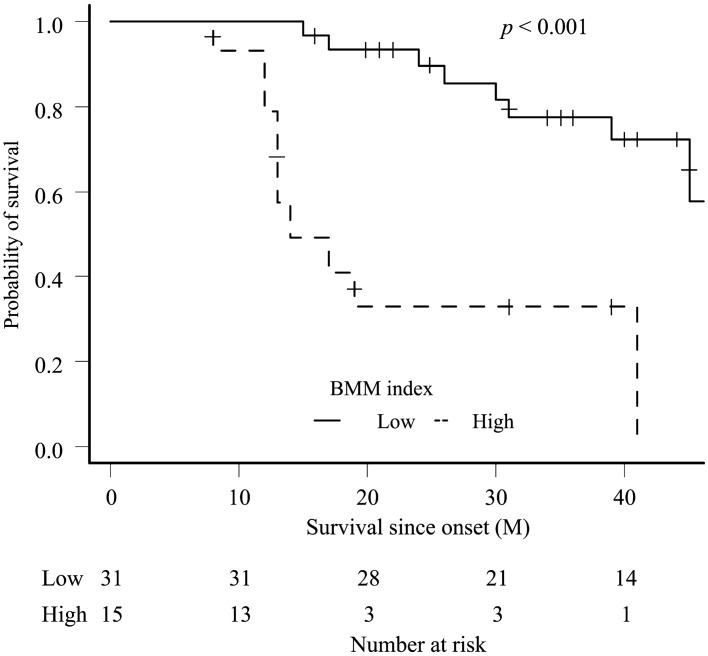
Comparison of survival rate between amyotrophic lateral sclerosis patients stratified by body mass index-muscle metabolism index (BMM index). The Kaplan–Meier analysis showed significant differences (log-rank test, *p* < 0.001).

In the high BMM index group, ΔALSFRS-R (*p* = 0.003) and ΔBMI (*p* = 0.004) were higher and lower than that in the low BMM index group, respectively. Regarding biomarkers, the high BMM index group had lower LDL (*p* = 0.011) and lower %VC (*p* = 0.002) compared with those of the low BMM index group. The Kaplan–Meier analysis and log-rank test revealed that the high BMM index group had significantly shorter survival time compared with that of the low BMM index group (p < 0.001) (Fig. 5). To examine the effect of the BMM index on the survival time since onset or this admission, we performed Cox proportional hazards (PH) model with the following confounding factors: age, ALSFRS-R, bulbar type, excess weight loss, high BMM Index, and %VC. Multivariate analysis showed BMM index and excess weight loss were independent poor prognostic factors (Table 3).

**Table 3:** Cox proportional hazards model.

		(A) Survival since onset		(B) Survival since this admission	
		Adjusted HR	p*	Adjusted HR	p*
BMM index	≥ 1 versus < 1	**4.05 (1.19**–**13.8)**	**0.025**	**5.90 (1.76**–**19.7)**	**0.004**
Age		0.98 (0.89–1.07)	0.66	0.99 (0.89–1.09)	0.80
ALSFRS-R		1.05 (0.94–1.17)	0.37	0.99 (0.90–1.09)	0.82
Excess weight loss^†^	Yes versus no	**3.07 (1.11**–**8.53)**	**0.031**	**2.95 (1.03**–**8.43)**	**0.043**
Bulbar type	Yes versus no	1.43 (0.52–4.00)	0.49	0.96 (0.30–3.01)	0.94
%VC		0.98 (0.94–1.02)	0.22	0.96 (0.92–1.00)	0.061

Bold font was used to strengthen the statistical significance.

*HR* Hazard ratio (95% confidence interval), *BMM* Body mass index-muscle metabolism, *ALSFRS-R* Revised amyotrophic lateral functional rating scale, *VC* Vital capacity.

*p value is based on Cox proportional hazards model.

^†^Excess weight loss means more than 10% compared to the body weight before the illness.

## Discussion

In this study we explored the effect of hypermetabolism on survival relative to the nutritional status of patients with ALS, and found that hypermetabolic patients with a normal-weight had shorter survival, whereas survival was longer in hypermetabolic patients with malnutrition. Of note, Steyn^10^ and He^11^ reported that hypermetabolism is a clear predictor of poor prognosis, and Bouteloup^9^ demonstrated similar results, although it was not statistically significant. Based on our results, the possible reason to explain these different statistics might be because the mean value of BMI in the Steyn paper was higher than that of the Bouteloup. The lower SMI and LSTM, a previously reported poor prognostic factor^13^ (Table 1), could be the reason the prognosis of our hypermetabolic normal-weight group is worse than that of other groups.

Surprisingly, hypermetabolism was a better prognostic factor in the malnutrition group (Fig. 1). Lipid biomarkers were characteristic in this group, which might contribute to the longer survival. The hypermetabolic malnutrition group had the highest median value of LDL among all the groups, and higher body fat percentage, which were good prognostic factors in previous studies. Indeed, patients with a high body fat percentage had longer survival in our study (Fig. 2), and the patients with high LDL showed a similar trend. It should be noted that the RQ was less than 0.85 in eight out of nine patients in this group (Table 1), and FBS was significantly higher in this group, which might indicate a lipid metabolism-shift from glucose assumption^34^. Indeed, the RQ was associated with ALSFRS-R and time since onset, and it is intriguing to hypothesize that a “fuel switch” may underlie metabolic profiles in ALS progression^4^. Furthermore, Steyn et, al. reported that high elevated fatty-acid oxidation increases whole-body energy expenditure and slows disease progression ^37^, which might support our results and indicate the contribution of hyper lipid metabolism for longer survival. Our findings caution against overestimation of hypermetabolism alone as a prognosis indicator.

Based on the possible involvement of skeletal muscles’ hypermetabolism in ALS prognosis^9,10^, we proposed the equation “BMM index = (BMI − 19.8) × (mREE/LSTM − 38),” which successfully predicted the prognostic factor, regardless of nutritional states. In agreement with a previous report^38^, our cohort demonstrated that weight loss of more than 10% was an independent poor prognostic factor. The rate of weight loss was faster in the high BMM index group; thus, the BMM index could be a valuable indicator to consider aggressive nutritional intervention, including PEG, at an early stage in clinical practice. High-fat or high-carbohydrate calorie supplementation is a matter of debate. Some reports suggested that high-fat calorie supplementation was a better nutritional treatment in ALS patients^39,40^, while others recommend high-carbohydrate calorie supplementation^41^. For patients with chronic obstructive pulmonary disease who have impaired CO_2_ emissions, a high-fat diet is recommended because lipid metabolism produces less carbon dioxide. Based on our result that lipid-shift trend might contribute a better prognosis, a high-fat intake could be an attractive option in a high-calorie diet for ALS. Of course, the choice of nutrition must be cautiously made, depending on the patients’ metabolic profiles.

The definition we adopted for hypermetabolism is a high mREE/LSTM, rather than the popular global index, mREE/pREE, because pREE estimated by the Harris-Benedict equation is reportedly not appropriate for the metabolic assessment of Japanese persons^29^. In our study, mREE/pREE positively correlated with LSTM, and thus mREE/pREE might be underestimated in patients with lower LSTM. Indeed, mREE/pREE was not found to be a beneficial prognostic factor in this study (Supplemental Fig. 2), contrary to previous reports^9,10^. On the other hand, mREE/LSTM was also negatively associated with LSTM in this study, which may cause overestimation of mREE/LSTM in patients with lower LSTM. However, high mREE/LSTM could reflect hypermetabolism more accurately than mREE/pREE in our cohort because mREE/LSTM was a poor prognostic factor in the normal-weight group, which was consistent with the previously reported fact that hypermetabolism was a poor prognostic factor^9,10^. There is no universally accepted index for assessing hypermetabolism, and mREE/LSTM may be a useful index for metabolic assessment in the Japanese population.

This study has several limitations. One is the small sample size; each nutritional group had a small number of patients. Second, this study is a retrospective study and included only Japanese patients in a single-center. The definition of malnutrition and hypermetabolism may be different in other countries, and it is not clear whether the prognostic prediction from mREE/LSTM depends on %IBW in other countries and this study. Adjusting the reference value of mREE/LSTM and BMI might make the BMM index more universally available. Of note, there are few studies on prognostic models for ALS in Asia, and this study is precious^42^. Third, we included patients at different stages with different reasons, such as close investigation, the introduction of therapeutic drugs and NPPV, and PEG. The validation of the timing of the BMM index in the clinical course is also required. Fourth, we did not perform a genetic test in most cases. Only one patient had the L106V mutation in the superoxide dismutase 1 (SOD1) gene, and this patient was in the hypermetabolic and normal-weight group and had a very high BMM index. The patient indeed showed a rapid progression. The relationship between genetic mutation and BMM index needs to be investigated in the future.

This is the first study to show that prognosis prediction by hypermetabolism varies depending on the nutrition status in patients with early ALS. The BMM index is a useful predictor of the prognosis, which could be referred to as the decision of interventions, such as PEG and NPPV, at an early stage to patients with a high BMM index.

## Supplementary Information

Supplementary Legends.

Supplementary Figures.

## Data Availability

The datasets generated during and/or analysed during the current study are available from the corresponding author on reasonable request.
