# Supplementary material for: Prognostic prediction by hypermetabolism varies depending on the nutritional status in early amyotrophic lateral sclerosis
Source: Sci Rep. 2021 Sep 9;11:17943. doi: 10.1038/s41598-021-97196-5 (PMC8429558; doi:10.1038/s41598-021-97196-5)
Supplement: Supplementary file 1 — Supplementary Legends. [file 41598_2021_97196_MOESM1_ESM.docx]

**Legends for supplemental figures**

To create figures, we used EZR (https://www.jichi.ac.jp/saitama-sct/SaitamaHP.files/statmedEN.html) and Microsoft Excel version 2016.

**Supplemental Figure 1** Comparison of survival time since this admission between the hypermetabolic group and the normal metabolic group in all patients (a), the normal-weight group (b), and the malnutrition group (c). Kaplan-Meier analyses and the log-rank test showed there was no difference in all patients (*p* = 0.37); however, the hypermetabolic group had a significantly shorter survival time in the normal-weight group (*p* < 0.001) and longer survival time in the malnutrition group (*p* = 0.17), although the difference was not significant.

**Supplemental Figure 2** Comparison of survival time since onset stratified by mREE/pREE in all patients (a), the normal-weight group (b), and the malnutrition group (c). Kaplan-Meier analyses and the log-rank test showed there was no difference.

**Supplemental Figure 3** Comparison of survival time since this admission between amyotrophic lateral sclerosis patients stratified by excess weight loss (a), body fat percentage (b), and low-density lipoprotein cholesterol (LDL) (c). Kaplan-Meier analyses and the log-rank test showed patients with excess weight loss (*p* = 0.007) and low LDL (*p* < 0.001) had significantly shorter survival time. Patients with low body fat percentage (*p* = 0.106) also had shorter survival time, although the difference was not significant.

**Supplemental Figure 4** The association between demographics (BMI, lean soft tissue mass [LSTM], and body fat percentage) and mREE/LSTM, mREE/pREE, and body fat percentage. Rs means Spearman's rank-correlation coefﬁcient. P-value is based on Spearman's test. mREE/LSTM was significantly correlated with BMI (a), LSTM(b), and body fat percentage (c). mREE/pREE significantly correlated with BMI(d), LSTM (e) and body fat percentage (f). Body fat percentage was significantly correlated with BMI (g) and LSTM (h).

**Supplemental Figure 5** Comparison of survival time since this admission between amyotrophic lateral sclerosis patients stratified by body mass index-muscle metabolism index (BMM index). The Kaplan-Meier analysis showed significant differences (log-rank test, *p* < 0.001).
