# Supplementary material for: Prognostic prediction by hypermetabolism varies depending on the nutritional status in early amyotrophic lateral sclerosis
Source: Sci Rep. 2021 Sep 9;11:17943. doi: 10.1038/s41598-021-97196-5 (PMC8429558; doi:10.1038/s41598-021-97196-5)

Supplemental information

Prognostic prediction by hypermetabolism varies depending on the nutritional status in early amyotrophic lateral sclerosis.

Ryutaro Nakamura, MD[1]; Mika Kurihara, MS[2], Nobuhiro Ogawa, MD, PhD[1]; Akihiro Kitamura, MD, PhD[1]; Isamu Yamakawa, MD, PhD[1]; Shigeki, Bamba, MD, PhD[2]; Mitsuru Sanada, MD, PhD[1]; Masaya Sasaki, MD, PhD[2], Makoto Urushitani, MD, PhD[1].

[1]Department of Neurology
[2]Division of Clinical Nutrition, Shiga University of Medical Science, Tsukinowa, Seta, Shiga, Japan

Supplemental Figure 1-5

Correspondence author
Makoto Urushitani, MD, Ph.D.
Department of Neurology, Shiga University of Medical Science, Tsukinowa, Seta, Shiga, Japan
Telephone/Fax: +81 (0) 77 548 2160
E-mail: uru@belle.shiga-med.ac.jp

Supplemental Figure 1

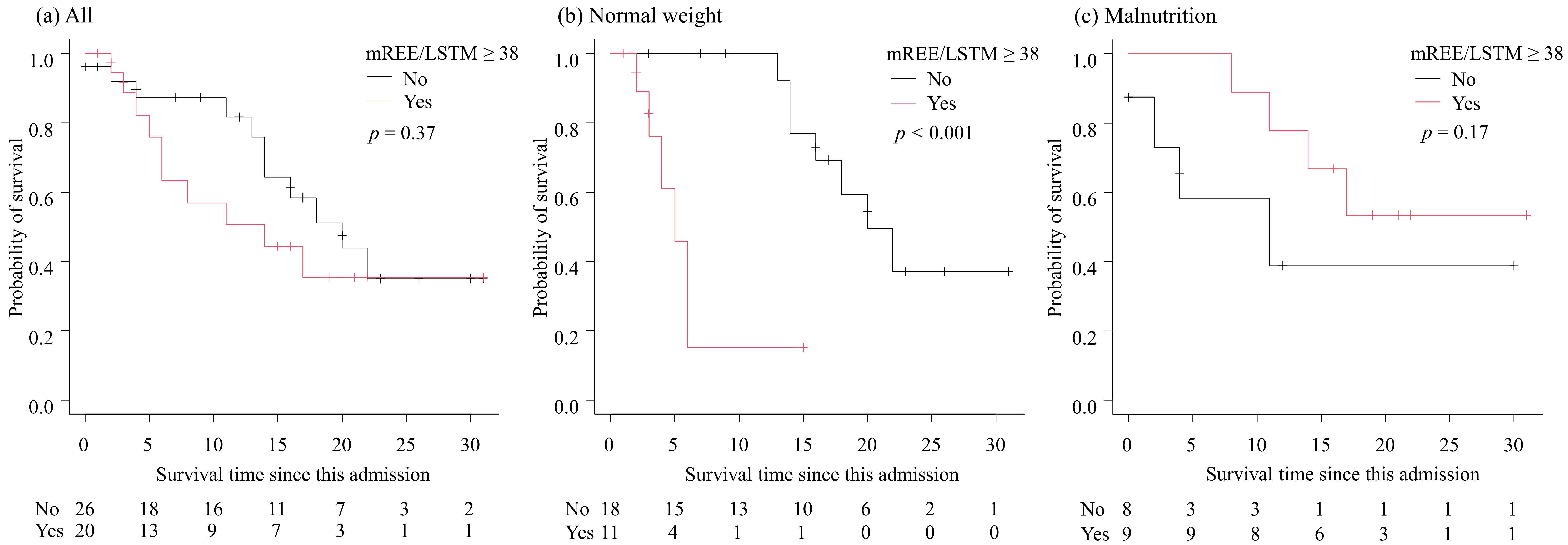

Supplemental Figure 2

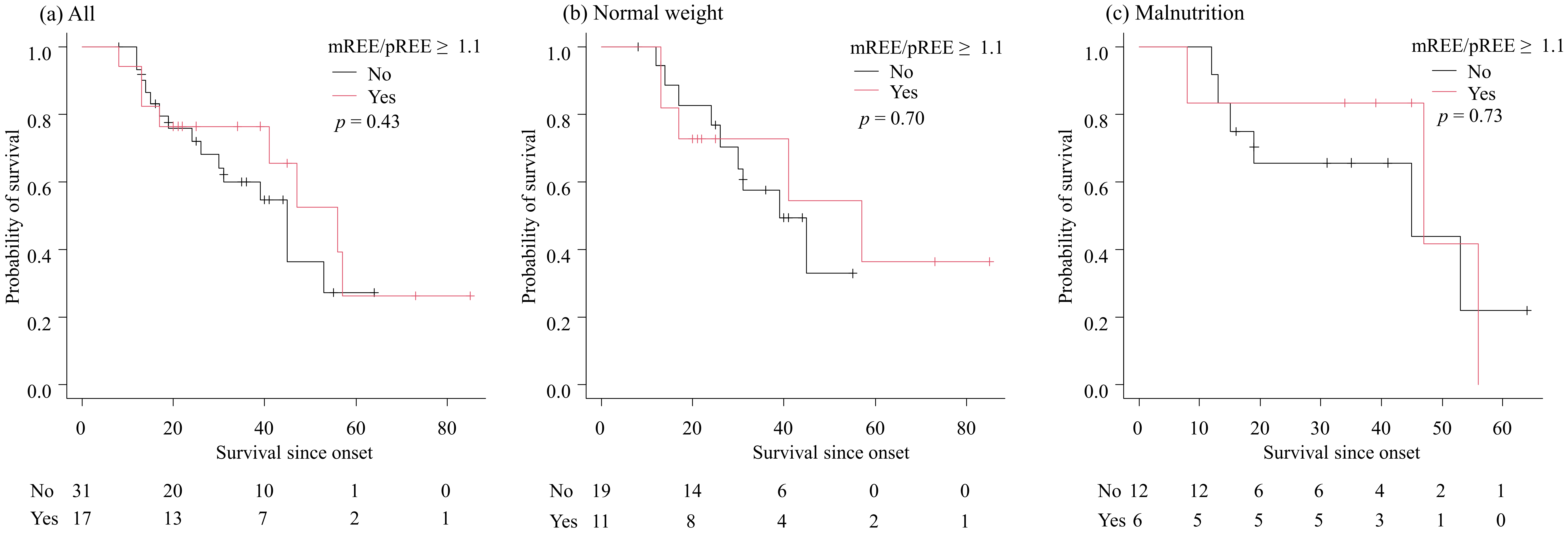

Supplemental Figure 3

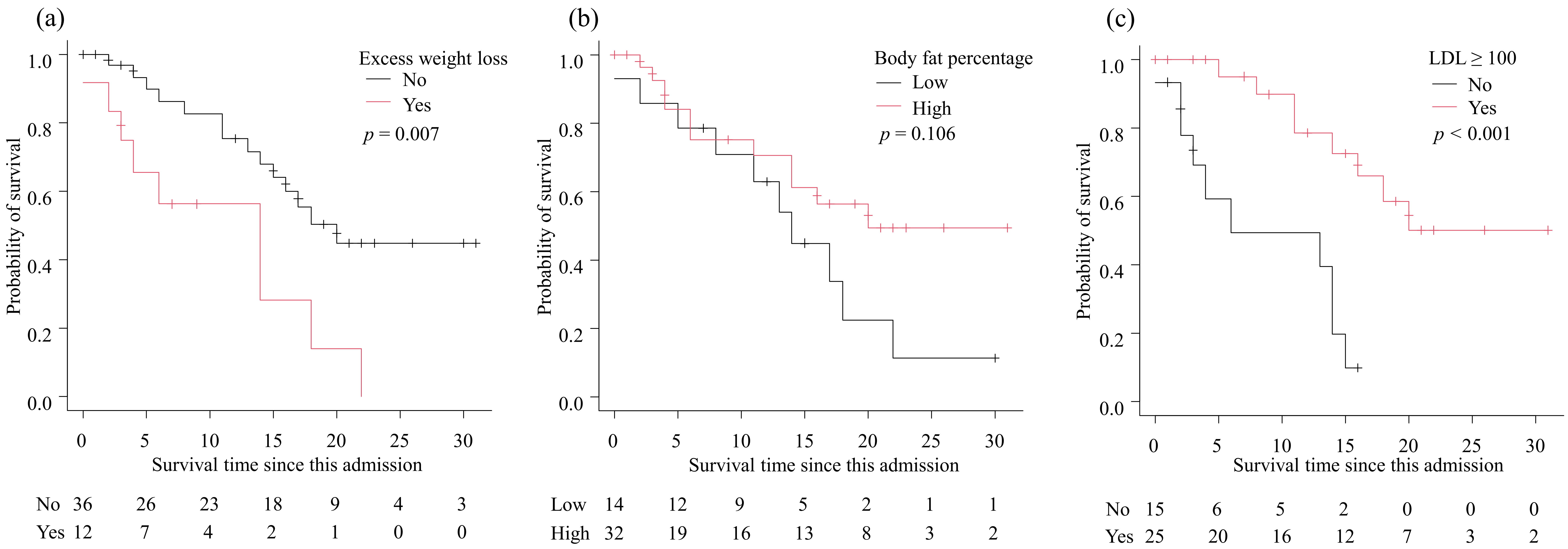

Supplemental Figure 4

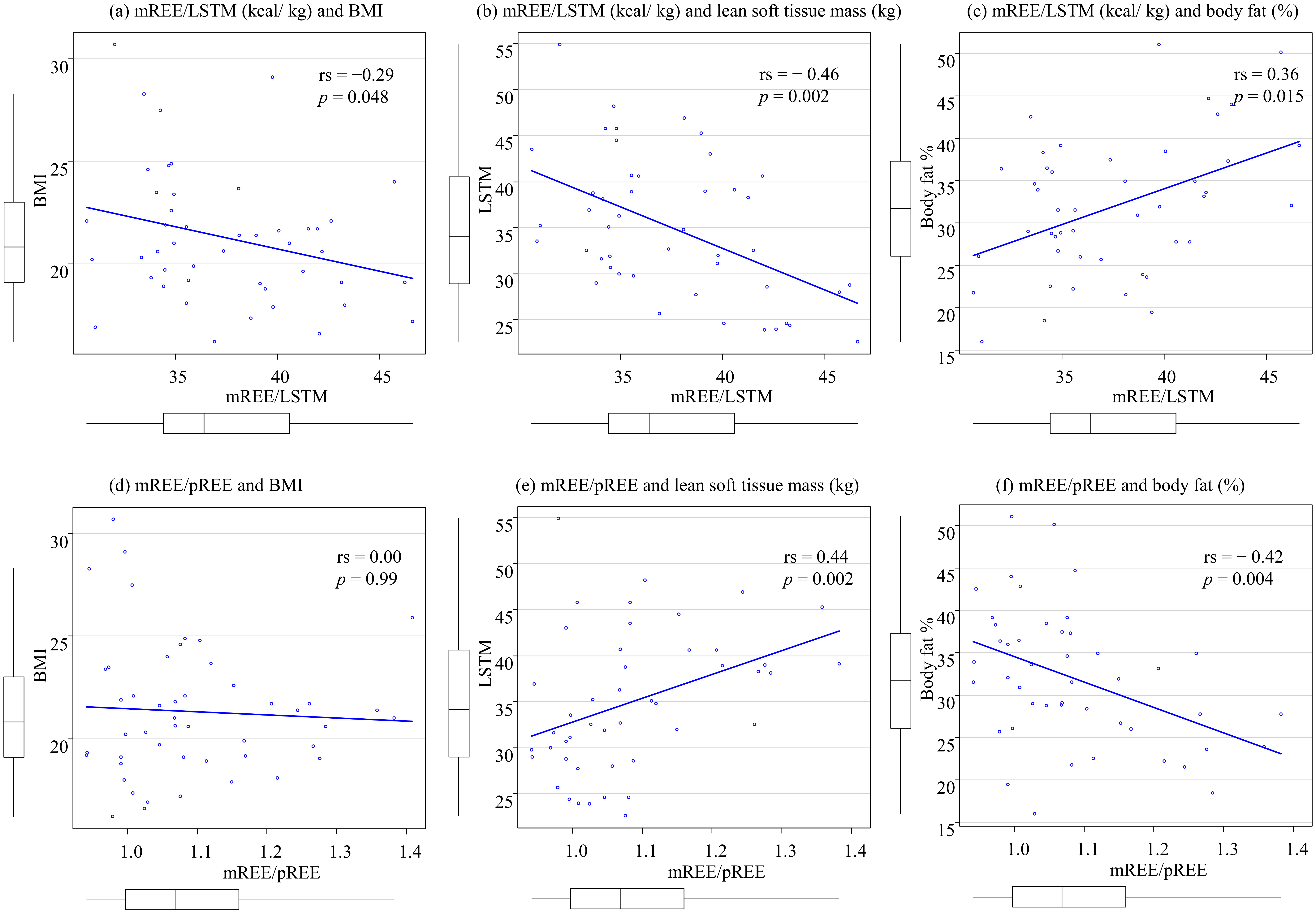

Supplemental Figure 4

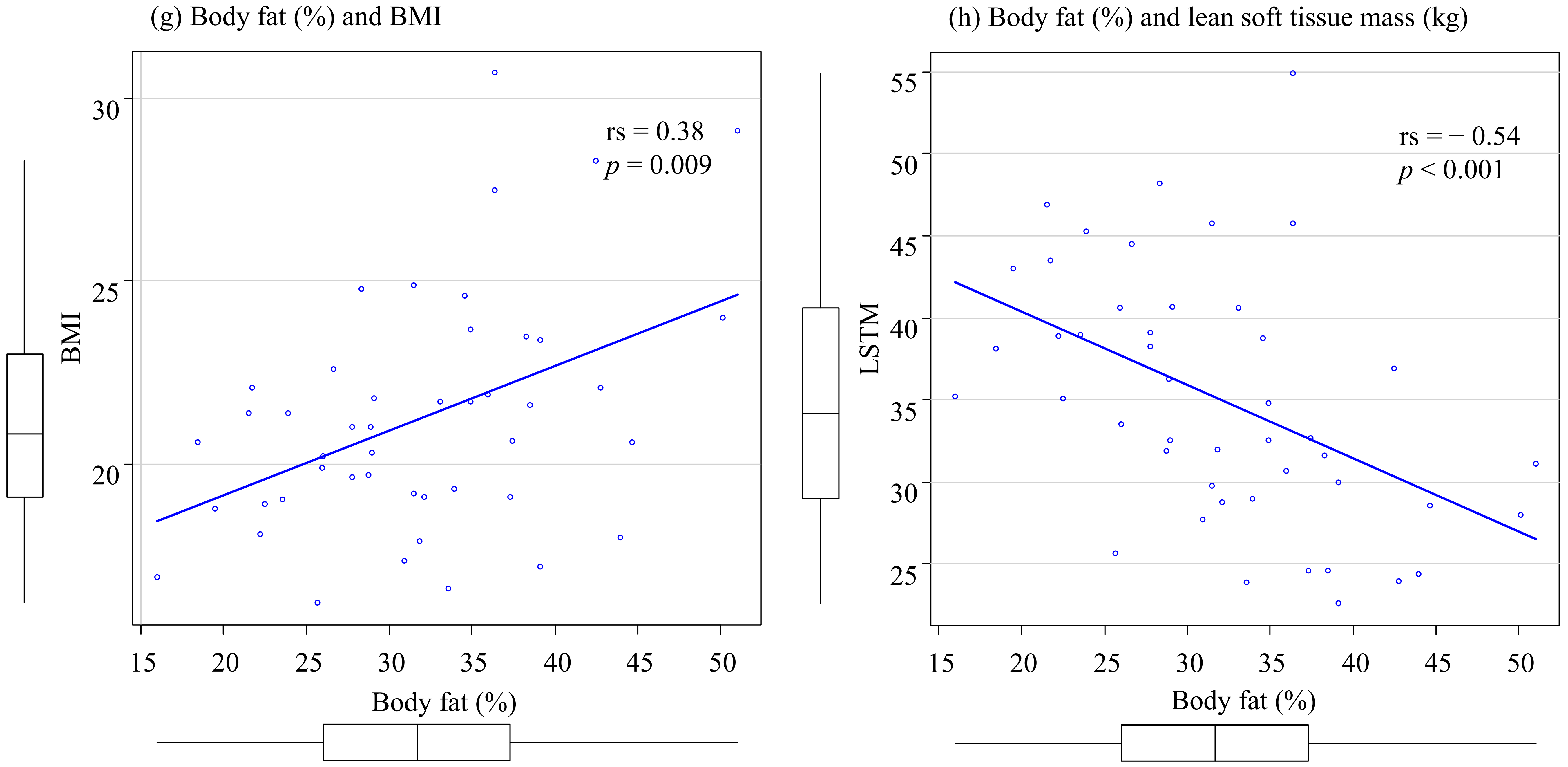

Supplemental Figure 5

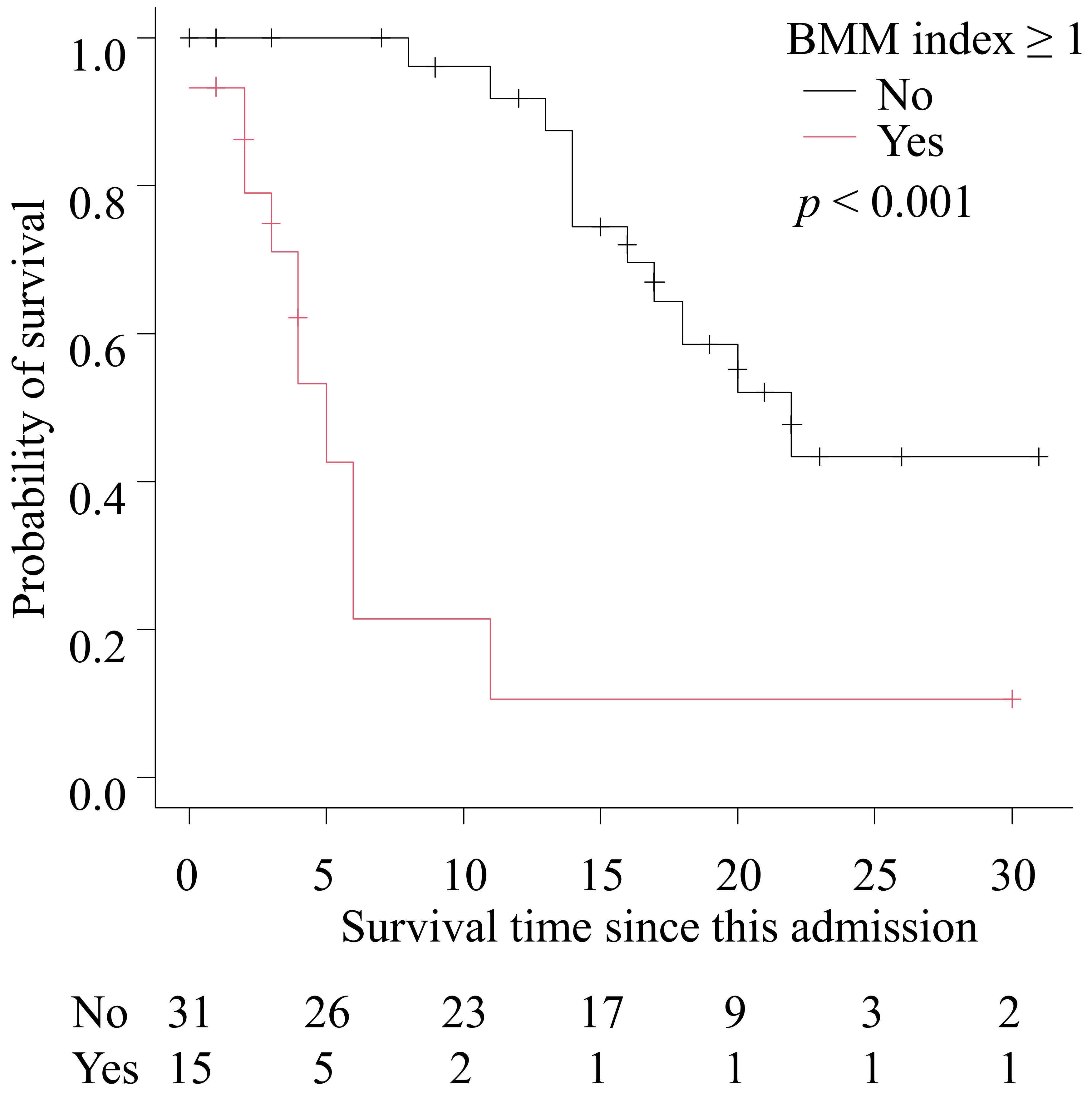

Supplement: Supplementary file 2 — Supplementary Figures. [file 41598_2021_97196_MOESM2_ESM.pdf]
